# Reimbursement of care does not equal the distribution of hospital resources: an explorative case study on a missing link among Dutch hospitals

**DOI:** 10.1186/s12913-023-09649-4

**Published:** 2023-09-19

**Authors:** L. V. L. van Leeuwen, R. Mesman, H. J. J. M. Berden, P. P. T. Jeurissen

**Affiliations:** Scientific Center for Quality of Healthcare, Radboud University Medical Center, P.O. Box 9101, 6500 HB Nijmegen, the Netherlands

**Keywords:** Reimbursement mechanisms, Budgets, Distribution model, Healthcare costs, Dutch, Incentives

## Abstract

**Background:**

Affordability and accessibility of hospital care are under pressure. Research on hospital care financing focuses primarily on incentives in the financial system outside the hospital. It is notable that little is known about (incentives in) internal funding in hospitals. Therefore, our study focuses on the budget allocation in hospitals: the distribution model. Based on our hypothesis that the reimbursement and distribution models in hospitals might interact, we gain knowledge about-, and insight into, the interaction of different reimbursement and distribution models used in Dutch hospitals, and how they affect the financial output of hospital care.

**Methods:**

An online survey with 22 questions was conducted among financial senior management as an expert group in 49 Dutch hospitals.

**Results:**

Ultimately, 38 of 49 approached experts fully completed the survey, which amounts to 78% of the hospitals we approached and 60% of all Dutch hospitals. The results on the reimbursement model indicate price * volume with adjusted prices above a maximum cap as the most common dominant contract type. On the internal distribution model, 75–80% of the experts reported incremental budgeting as the dominant budgeting method. Results on the interaction between the reimbursement and the distribution model show that both general and specific changes in contract agreements are only partially incorporated in hospital budgets. In 28 out of 31 hospitals with self-employed medical specialists, a relation is reported between the reimbursement model and the contracts with the Medical Consultant Group(s) in which the medical specialists are united.

**Conclusions:**

Our results in Dutch setting indicate a limited interaction between the reimbursement model and the distribution model. This lack of congruence between both models might limit the desired effects of incentives in contractual agreements aimed at the financial output. This applies to different reimbursement and distribution models. Further research into the various interactions and incentives, as visualized in our conceptual framework, could result in evidence-based advice for achieving affordable and accessible hospital care.

**Supplementary Information:**

The online version contains supplementary material available at 10.1186/s12913-023-09649-4.

## Background

Affordability and accessibility of hospital care are under pressure, caused in part by rising costs. In 2020, the global health expenditure reached US $9 trillion (approximately 11% of global Gross Domestic Product) [1]. Hospital care is a significant component of health care spending. Controlling these costs, requires attention on health care financing. A global overview of the comprehensive literature on financing hospital care shows a variety of financial systems worldwide. Differences may be seen in sources of funding (e.g. public or private funding) and reimbursement mechanisms (e.g. activity-based funding or cost-based payment models) [2]. Research on hospital care financing focuses primarily on incentives in the financial system outside the hospital. It is notable that little is known about (incentives in) internal funding in hospitals. Therefore, our study focuses on the budget allocation in hospitals: the distribution model.

The financial model for hospitals can be separated into two components, the reimbursement and the distribution model. The reimbursement model relates to revenue for the hospital. In the reimbursement model, contracts with health insurers contain incentives that might influence costs of hospital care [3, 4]. Incentives are incorporated into reimbursement models to influence the amount of care delivered in hospitals. In doing so, the contracting party attempts to control the hospital's (financial) output [5]. Different contract types are described in Table 1. Hospital distribution models determine the actual financial space available for a department in a hospital to provide care. Budgets in hospitals are allocated annually to departments, this refers to the distribution model of hospitals. How budgets are distributed in hospitals can vary as further explained in Table 1. From a strategic/managerial perspective, the distribution of budgets within the hospital should reflect the organizational goals [6]. Based on available budgets, departments can provide care. The more budget available, the more personnel and equipment available to provide care. Therefore, the distribution model contains incentives to provide care. These incentives are not always consistent with the content of the contract agreements in the reimbursement model [7].

**Table 1:** Contract types and budgeting methods

Contract types in this study, ranked in order of level of incentive to deliver treatments (low to high):
– **Lump sum agreement:** revenues received regardless of the amount of care provided
– Price * volume (P*Q) agreement with a maximum cap: until the cap is reached, more treatments result in more revenue. If the cap is reached, additional care is not reimbursed
– P*Q agreement with adjusted prices above a maximum cap: until the cap is reached, more treatments result in more revenue. If the cap is reached, additional care is reimbursed at a reduced rate
– P*Q agreement without a maximum cap: more treatments result in more revenue
The contract types in this study are ideal–typical; in practice, mixed variations are used. In 2020, the most common contract types between health insurers and Dutch hospitals were lump sum agreements, agreements without a maximum cap, and agreements with a maximum cap [8]
Budgeting methods in this study, ranked in order of level of adaptive capacity (high to low):
– **Activity-based budgeting:** budget based on actual calculated costs and expected production
– **Direct budget distribution:** direct allocation of budget for (one-time) investments or other specific (one-time) expenses
– **Incremental budgeting:** budget equal to the previous year (history-based) with possible correction for indexing and specific changes in provided care

Incentives may be present in both the reimbursement model and the internal and external distribution model that affect hospitals' financial output. The relationship between costs and revenues in hospitals is complex. Cross-subsidization allows for both positive and negative margins on care provided. Therefore, there is often no direct relationship between revenues generated and costs incurred by the hospital [9]. Standardized rules for calculating costs of hospital care are often not available. Hospitals may choose for differences in calculation methodologies for their costs. On top of structural varieties this further reduces the knowledge about actual costs [10]. Different choices in allocation reduce transparency in actual costs and therefore it reduces the transparency of the relation between costs and revenues. Variation in hospital costs is associated with the contractual arrangements between hospitals and health insurers [3].

Internationally, affordability and accessibility of care are under pressure. We assume that both the reimbursement and the distribution model do contribute to the financial output of hospitals. Thus, both financial models affect the affordability and accessibility of care. This holds for all health care systems. However, despite its relevance, little is known about the distribution model within hospitals. Policymakers and researchers mainly focus on incentives in the hospital reimbursement model while the internal budget distribution of hospital resources remains largely unknown [3, 4, 11]. We assume this is because of limited transparency about the allocation of budgets (the distribution model) within hospitals [12]. Table 1 details the most common budget models. Contracts between hospitals and health insurers contain agreements regarding financial output such as number of treatments and revenue for the hospital. However, reimbursement and distribution models in hospitals might interact and this may either limit or exceed the working of the formal reimbursement mechanisms. Therefore, we consider the distribution model as a missing link in the understanding of the conversion of the reimbursement model to the financial output of hospital care. This study aims to provide insight into the distribution model as well as the possible relationship with the reimbursement model. We hypothesize that the interaction between the reimbursement model and distribution model in hospitals affects the financial output of hospital care.

To examine our hypothesis, we use the Netherlands as a case study. In 2021, total healthcare costs were expected to be 11,2% of the Gross Domestic Product in the Netherlands [13]. This is consistent with the global healthcare expenditure. Hospital care in the Netherlands represents 30% of total healthcare costs [14] and is reimbursed through a mechanism in which health insurers act as a third-party payer [15]. One reason why this case holds special interest may be the wide variety in contract arrangements for reimbursement as well as a high degree of flexibility in structure and budgeting. This may lead to interesting comparisons. Relevant information about the financing system in Dutch hospitals is provided in Table 2. Insights gained in this case study are applicable to various reimbursement and distribution models of hospitals in other countries.

**Table 2:** Financing system in Dutch hospitals

Since 2005, hospital care in the Netherlands is financed by DTCs (Diagnosis Treatment Combinations, translated from Dutch: Diagnose-BehandelCombinatie or DBC), that cover all costs for treatments [16, 17]. The DTC system has similarities to a detailed DRG system. Like the DRG system, the DTC system aims to increase transparency and efficiency and use financial incentives to reduce (unnecessary) use of care [18]. Unlike the DRG system, a DTC covers both inpatient and outpatient hospital care and simultaneously multiple DTCs can be recorded for different treatments [19]. Insurance companies and hospital providers have substantial leeway to negotiate reimbursements. Since 2012 70% of the DTC prices are freely negotiable between hospitals and health insurers as a third party purchaser [20]. The classification in so-called DTCs does take place according to a national system but all health insurers negotiate specific prices with all hospitals on DTCs. This typically result in different pricing for the same treatment between hospitals, but also different pricing within one hospital depending on the paying health insurer. The large variation in claim prices between both Dutch hospitals and insurance companies confirm that claims do not reflect actual costs [10]. In addition, contracts may vary in duration, size, and contract type. Different contract types are clarified in Table 1
Three types of hospitals are distinguished in the Netherlands: 7 University Medical Centers (UMCs) that combine teaching, research and highly complex care [21]. 27 Teaching hospitals (THs) with focus on top-clinical care nearby [22] and 29 General hospitals (GHs) with focus on primary care [23]. The employment of medical specialists varies in each hospital. In some hospitals, for example all UMCs, all medical specialists are employed by the hospital. In THs and GHs, 65% of the medical specialists are self-employed [24]. In the Netherlands these medical specialists are united in associations, so called Medical Consultant Groups (MCGs). An MCG, on behalf of all its affiliated self-employed medical specialists, has an agreement with a hospital regarding the care to be provided and the budget. This agreement concerns the external distribution model. It is possible that there are several MCGs associated with one hospital [24]. Financial incentives for medical specialists differ depending on their employment. Medical specialists employed by a hospital do not have a production incentive with a direct relationship to their income, although the budget distribution within the hospital (internal distribution model) contains incentives that could affect their production. In MCGs, income distribution between medical specialists usually contains a direct incentive to provide care [25]

## Methods

### Conceptual framework

We developed a conceptual framework to visualize the financing structure of hospitals and explore the relation between the financing structure and the financial output in Dutch hospitals.

**Fig. 1:**
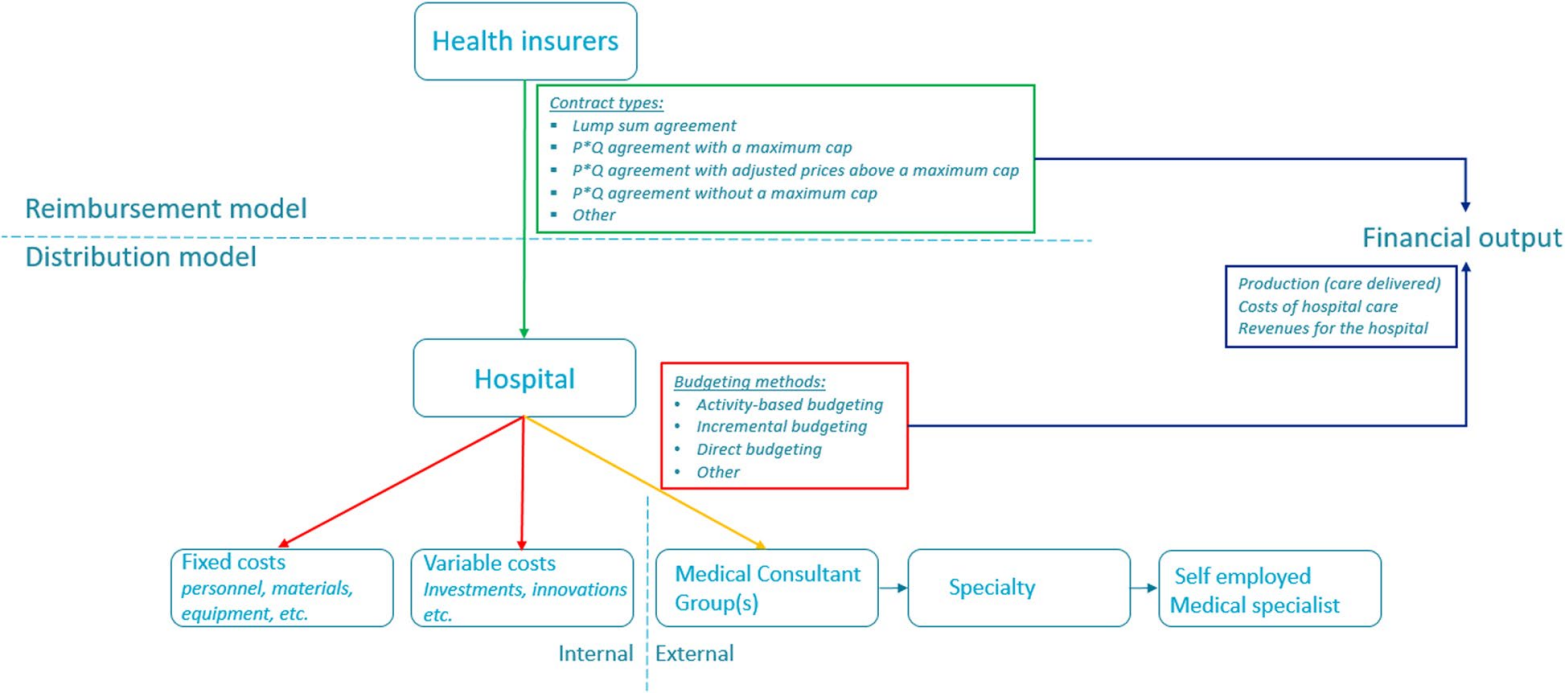
Conceptual framework of the financial model for hospitals

Figure 1 contains our conceptual framework which consists of three parts. The upper part (green arrow) represents the reimbursement model of a hospital and is related to the contract agreements with health insurers. The bottom part shows the internal- (red arrows) and external (orange arrow) distribution model of a hospital, which refers to the annual budget allocation. The part on the right-hand side (dark blue arrows) indicates the financial output generated by hospitals, e.g. production (care delivered), costs, and revenues. We consider financial output as a function of both the reimbursement and the distribution model. The arrows in the conceptual framework represent the dispersion of finances in the system, which can contain different financial incentives. The actual strength of these incentives depends on the methods used in de reimbursement and distribution model and their interaction. Table 1 illustrates the different contract types and budgeting methods.

### Survey

We designed a questionnaire that is based on our conceptual framework and aims to explore the hypothesis that the interaction between the reimbursement model and distribution model in Dutch hospitals affects the financial output of hospital care. The questionnaire is included in Supplementary 1.

Our draft questionnaire was tested by one financial manager and one senior financial controller. These pilots resulted in minor changes in terminology and sequence of questions. The survey consists of a total of 22 questions. The survey was available for completion from March 21 until April 1, 2022; the deadline was eventually extended to May 3, 2022 to gain more responses.

The questionnaire consists of four sections. 1) seven general questions to ensure the appropriateness of the expert and to collect characteristics of the hospital. 2) eight follow-up questions to provide insight into the characteristics of the contract types and budgeting systems used in different hospitals. In a world of ideal-type budgeting, the most flexible method is activity-based budgeting, the most fixed method is incremental budgeting [6]. In this survey, we included these two contrasting budgeting methods. In addition, we added direct budget distribution as an intermediate method. This method is mainly used for investments where budgets are allocated directly. For example, budget for the purchasing of medical appliances. 3) four questions to generate insight into the relation between the reimbursement model and the distribution model. To examine whether a change in the reimbursement model is incorporated into the distribution model, we provided two examples (question 16 and 17). The internal distribution model and the external distribution model are examined separately. 4) three questions about willingness to participate in follow-up research.

### Sample selection/participants and data collection

A thorough theoretical and practical knowledge of both the reimbursement model and the distribution model of the hospital is required for answering the questionnaire. Therefore, the choice was made to approach experts (financial managers) of hospitals. Approaching all financial experts in hospitals turned out to be not feasible, mainly due to practical considerations. We chose to make the group as large as possible with a representation of as many hospitals as possible, sufficient representing all three hospital categories (UMCs, THs, GHs). We approached 49 financial experts, most of them directly by someone from the research group. Eight financial experts were contacted through the Dutch association of professionals in healthcare finance (Fizi).

The questionnaire was sent as a link by email to 49 financial managers in Dutch hospitals in March 2022. Participants received up to three reminders in March and April 2022. All results were processed directly in LimeSurvey. Dummy codes were created for participants who were approached indirectly by Fizi. In those cases, the questionnaire and reminder messages were sent by email to Fizi. Contradictory answers occurred in questions 8b and 14b (see Supplementary 1 for the survey). These questions asked for the extent of budget systems and contract types used. Collectively, the answers in these questions should add up to 100%. For 15 participants, this was not the case. We contacted these participants by email and allowed to provide a corrected answer which was included in the results. In 3 cases, no response to the e-mail was obtained. This resulted in 3 conflicting responses to question 8b (see Supplementary 1 for the survey). In all 3 cases, the sum of the answers exceeded 100%. The response options to this question consisted of ranges. Based on the average value in this range, we determined the total percentage as completed by the respondent. We redistributed this total proportionally so that the total added up to 100%. We then converted this division to the ranges as given in the answer options in question 8b. Finally, we cross-checked the weighted redistribution with the results for question 8a where the budget systems were ranked. Finally, some open-ended questions were asked to provide further clarification. The answers to these questions were thematically grouped.

### Data analysis

The data collected in LimeSurvey was exported into Microsoft Office Excel 2016 for data analysis. Due to the limited number of hospitals in the Netherlands and consequential small sample size, it was not possible to obtain statistically significant results. Therefore, results are presented using descriptive statistics. Since there were no outliers due to the response options in the multiple-choice questions, percentages reflect a good representation of the ratio between the responses. The dominant contract type and budget method are based on the percentages that were provided in questions 8b and 14b (Supplementary 1). When two results emerged with the highest percentage, the optional ranking questions 8a and 14a (Supplementary 1) were used to assess the dominant system. Answers to the open-ended questions are presented narratively.

## Results

### General information

Ultimately, 38 of 49 approached experts fully completed the survey, which amounts to 78% of the hospitals we approached and 60% of all Dutch hospitals (Supplementary 2, Table S1). One partially completed questionnaire was excluded from the analysis. Experts in 5 out of 7 UMCs were approached to participate and all of them completed the questionnaire. All Dutch THs were contacted and 89% completed the questionnaire. Based on available contact data of experts, 17 of the 29 GHs in the Netherlands were approached, of which 9 experts participated in this study. Supplementary 2, Table S2 shows an overview of the number of Medical Consultant Groups (MCGs) related to the hospitals in this study. As mentioned in Table 2, MCGs are not related to UMCs. Notably, over 60% of the THs have one related MCG and almost 80% of the GHs have multiple related MCGs.

### Reimbursement model

The market share of the largest health insurer in terms of the total revenue to be contracted is > 50% in 12 hospitals. In 4 hospitals, the market share of the largest two health insurers combined does not exceed 50% of the total revenue of the hospital. The length of the contract with the health insurer with the largest market share is shown in Table 3. A one-year contract agreement with the health insurer with the largest market share is agreed upon by 24 out of 38 hospitals (63%), whereas 4 hospitals (11%) negotiate contracts for a period of five years or more.

**Table 3:** Characteristics of contracts with health insurers

Dominant contract type	Contract duration with the health insurer having the largest market share					
	1 year	2 years	3 years	4 years	5 or more years	**Total**
Lump sum	2	1	3	1	3	**10**
P*Q with a maximum cap	7	1			1	**9**
P*Q with adjusted prices above a maximum cap	13		1	2		**16**
P*Q without a maximum cap						**0**
Other	2			1		**3**
**Total**	**24**	**2**	**4**	**4**	**4**	**38**

It is common to combine different contract types within a contract between a hospital and a health insurer. Furthermore, contracts are agreed upon with different health insurers. Therefore, multiple contract types can be applied per hospital (Supplementary 3, Fig. S2). In our sample, no hospital indicated using only one contract type. The most frequently chosen contract type (33 hospitals) is P*Q with adjusted prices above a maximum cap. A dominant contract type for over 80% of the total contract scope of the hospital is used in 18 out of 38 hospitals (47%). When contract type "other" was chosen, the explanations provided by the experts mostly revealed use of a variation on the most common contract types.

It is noticeable that hospitals often use more than one contract type. Given the complexity of hospital care financing, for the purpose of our study we have chosen to focus on the possible interaction between the dominant contract type and the health insurer with the largest market share. As shown in Table 3, P*Q with adjusted prices above a maximum cap is the most common dominant contract type. This is followed by lump sum contracts and P*Q with a maximum cap. These contract types were mainly used in one-year contracts. When a contract for two or more years is agreed upon, lump sum is the most common dominant contract type. Agreements without a maximum cap were not dominant in any hospital.

### Distribution model

Hospitals annually distribute budgets to their departments. As presented in Table 1, there are different methods for the allocation of budgets. Equal to contract types, hospitals apply different budgeting methods simultaneously (Fig. S3 in Supplementary 3). All hospitals use incremental budgeting to some extent. In 17 hospitals, one budget type is used for over 80% of the internal budget distributions. In 16 of these 17 cases, the experts report incremental budgeting. In the remaining hospital activity-based budgeting is applicable. Direct budget distribution and activity-based budgeting are applied in respectively 30 and 31 hospitals, but most often not as the dominant method. If a hospital has opted for "other" budget method, an explanation is given of the specific characteristics. Other budget methods were used in 7 hospitals, but only to distribute a limited part of the budget. They mainly consisted of a variant of the ideal–typical methods presented in this study.

Hospitals use different budget methods to some extent. To avoid this complexity distracting from the purpose of our study, we focus on the dominant budget method in the results. Table 4 shows the dominant budget method for each type of hospital. Incremental budgeting is indicated in 30 out of 38 hospitals as the dominant method to distribute resources, which includes all UMCs. Activity-based budgeting is indicated as the dominant method in 6 THs (25%).

**Table 4:** Dominant budget methods per type of hospital

	**Hospital type**			
**Dominant budget method**	UMC	TH	GH	**Total**
Activity-based budgeting	0	6	1	**7**
Direct budget distribution	0	1	0	**1**
Incremental budgeting	5	17	8	**30**
**Total**	**5**	**24**	**9**	**38**

### Interaction between the reimbursement and distribution model

Table 5 provides insight into the dominant contract types and budget methods. The most common combination is incremental budgeting and P*Q with adjusted prices above a maximum cap. Irrespective of the dominant contract type, 75–80% of hospitals relied on incremental budgeting.

**Table 5:** Dominant contract types and budget methods

	**Activity-based Budgeting**	**Direct budget distribution**	**Incremental budgeting**	**Total**
Lump sum	2		8	**10**
P*Q with a maximum cap	2		7	**9**
P*Q with adjusted prices above a maximum cap	3	1	12	**16**
P*Q without a maximum cap				**0**
Other			3	**3**
**Total**	**7**	**1**	**30**	**38**

We examined whether a change in the reimbursement model is incorporated into the distribution model. In the questionnaire, we made a distinction between 1) a generic budgetary change related to the entire hospital in the contract agreement (Supplementary 1, question 16) and 2) a specific change related to one specialty (ophthalmology) in the contract agreement (Supplementary 1, question 17). Results are shown in Table 6.

**Table 6:** Relation between the reimbursement model and the distribution model

	**No. of hospitals**	**Percentage**
**Incorporation of a generic contract change in the budget distribution**	**38**	
Direct relation (fully incorporated in accordance with contract change)	6	15,8%
Indirect relation (partially incorporated in accordance with contract change)	27	71,1%
No relation (not incorporated in accordance with contract change)	5	13,2%
**Incorporation of a specific contract change in the budget distribution**	**38**	
Direct relation (fully incorporated in accordance with contract change)	18	47,4%
Indirect relation (partially incorporated in accordance with contract change)	13	34,2%
No relation (not incorporated in accordance to contract change)	7	18,4%

In case of a generic change in the contract agreements, 6 hospitals stated that they would fully adopt the change in their distribution model. In the majority of the hospitals (71%), there is an indirect relation between the change in the contract agreements and the budget distribution. In question 16a, where an explanation of this relationship is required, experts report strategy, policy, and market development as explanations for not incorporating the change fully into the distribution model. When a specific change in the reimbursement model is suggested, 18 hospitals (47%) stated that they would fully incorporate the change in their distribution model (Table 6). The number of hospitals indicating no connection at all is 7 (18%). In case of a direct adaptation, the most common explanations provided in question 17a are: being consistent, assigning a specific change, and avoiding incongruity. In case of an indirect relation, the results in question 17a indicate that only the marginal/variable costs are allocated specifically. For the absence of a direct relation in both a generic change and a specific change, experts mention timing and the absence of equality between the internal- and the external system as relevant factors.

Finally, hospitals with one or more MCGs were asked how a change in the reimbursement model is reflected in contract agreements with MCG(s). A relation between the reimbursement and external distribution models is indicated in 28 of the 31 hospitals with one or more MCGs (Table 7).

**Table 7:** Relation between the reimbursement model and contract agreements with the MCG(s)

	**Number of Medical Consultant Groups**				
	**0**	**1**	**2–3**	** > 3**	**Total**
No relation	7	2	1	0	**10**
A relation, but not with all MCGs	0	0	3	1	**4**
A relation with all MCGs	0	14	9	1	**24**
**Total**	**7**	**16**	**13**	**2**	**38**

## Discussion

Rising healthcare costs are putting pressure on the affordability of hospital care. In efforts to better understand and contain this development, both researchers and policy makers are primarily focused on the reimbursement model. However, an absolute focus on the reimbursement model provides a narrow view on the complex puzzle of hospital care financing and its incentives. By conducting a survey aimed at senior financial management in Dutch hospitals, we can shed some light onto a frequently overlooked mechanism: the interaction between the reimbursement model and budget distribution.

### Reimbursement model

We have now also reflected this in the discussion paragraph: Hospitals have contract agreements with multiple health insurers. These agreements may differ and thus may contain different incentives. As a result, the hospital has a mixture of funding sources [26]. To reduce complexity, in this study we focused on the contract with the health insurer with the largest market share. In most hospitals in this study, a contract duration of one year is agreed upon with the health insurer who represents the largest market share. For these hospitals, contract types can change annually which might cause fluctuations in revenue and limits long-term continuity. Long-term contracts between hospitals and health insurers can be a sign of trust [27]. Although long-term contracts can provide financial stability in terms of revenue, prices and volumes are not fixed for the entire duration of the contract [28]. Thereby, the hospital will have to continue to meet indicators for example in terms of quality and waiting time. All hospitals in this study maintain more than one contract type. As a result, incentives in the reimbursement model vary within and between different contracts, which can be prohibitive for implementing policy changes on appropriate care. Appropriate care is the collective term for the goal of the current Dutch government to ensure good care in the future that is proven effective and with a focus on health and prevention, nearby the patient if possible and together with the patient (shared-decision making) [29, 30].

Despite the diversity in contract type and duration, P*Q with adjusted prices above a maximum cap are the most used contract type in our sample. Especially when one-year contracts are agreed upon. This contract type contains substantial financial incentives to deliver more treatments. Some regulators recommend to abolish this contract type in order to avoid unnecessary care [31]. By contrast, a lump sum contract with limited incentives contributes to lower growth in intensity of care and offers more financial certainty for hospitals [26, 32]. Among long-term contracts, lump sum is the dominant contract type. It is stated that lump sum contracts create financial security to implement changes on appropriate care, for example to lower volumes. However, this creates the risk of the ratchet effect at the end of the multi-year contract [33]. It might no longer be possible to return to agreements prior to the multi-year agreement.

### Distribution model

Although different methods are used for budget distribution in hospitals, incremental budgeting is applied in all hospitals in this study. The limited available literature confirms our finding [6, 34]. Incremental budgeting is a fixed method based embedded in history [6]. As a result, the ability to adjust budgets based on changes in strategy is limited. Therefore, the budget often does not align with the current situation and strategy within a hospital [9]. Hospitals use the more flexible budgeting methods -such as direct budget distribution and activity-based budgeting- mainly for a limited part of the budget distribution.

### Interaction between the reimbursement and distribution model

To identify the extent to which financial incentives in the contract arrangements affect the hospitals financial output, it is relevant to look at the budget distribution within the hospital. In long-term contracts, a reimbursement model with few incentives to deliver care is often used in combination with a fixed budget method. One of the main reasons for agreeing on a long-term agreement with few incentives to deliver care is to restrain growth in care by preventing unnecessary care. This could contribute to accessibility of care. While lump sum contracts may indeed help to reduce treatment intensity, it may also contribute to an increased number of patients [26]. Another reason is to provide flexibility for change [28]. However, by using a fixed budget method such as incremental budgeting, budget distribution within the hospital may not change in accordance with change in the reimbursement model.

In contrast, in one-year contracts, reimbursement models with more financial incentives to provide care are dominant. Flexible budgeting methods are used more often than in long-term contracts, but still to a limited extent. This implies that if a contract contains more incentives to produce care, an adjustment in the budget distribution may occur more frequently. Short-term contract agreements with incentives to deliver more treatments, complicate changes in care such as cooperation between healthcare providers [35]. In many developed countries, the revenue model of hospitals contains incentives to affect financial output [5]. Both activity-based financing models (for example with Diagnostic-Related Groups) and cost-based financing models (such as Fee-For-Service) used in most countries contain incentives that might influence financial output [36]. Either to deliver more care per patient or to treat more patients. For a hospital, the diversity in financial incentives to produce care in the reimbursement models complicates managing financial output. A flexible budget distribution within the hospital might help to control the financial output.

Regardless of contract length, contracts between Dutch hospitals and health insurers are relatively high in financial incentives while the dominant budget method is rather rigid. This may explain limited incorporation of generic changes in hospital contract arrangements. In the event of a specific change, hospital budgets are more likely to be adjusted according to the change. However, still in less than half of the hospitals a specific change in the contract arrangements is fully implemented in the budget distribution. When the budget distribution model does not (fully) incorporate the incentives built into the reimbursement model, no behavioral change can be expected from professionals. Hospital reimbursement is highly complex. The average hospitals may conduct thousands of procedures. As a consequence cross-subsidies are omnipresent. Hospitals hold positive margins on certain procedures and negative margins on other procedures. This is substantial and even hospital administration may be unaware on a part of these cross-subsidies. Therefore, changing the production profile can have a different effect on revenues versus costs. In addition, in the highly regulated area of healthcare the marginal revenues (MR) of the reimbursement models may not equal marginal costs (MC). Fixed costs, such as salaries and building expenses, persist regardless of production volume. Changing fixed costs takes time. Multi-year agreements with certainty about revenues provide flexibility to adjust fixed costs [37]. As a result, pricing is typically not economically optimal and thus perverse incentives can arise from this. High hopes that seek to increase efficiency by reducing low-value and thus cost care may fail [38]. To our knowledge this holds for many countries.

Finally, we examined the typically Dutch phenomenon of MCGs and their contractual arrangements with hospitals (external distribution model). Nearly all MCGs are involved in the contract negotiations between the hospital and the health insurer [39]. Therefore, they have an active role in the composition of the reimbursement model. It is notable that almost all hospitals with MCGs indicate that there is a relationship between a change in contract arrangements with health insurers and the arrangements with the MCG(s). Contracts regarding the external distribution model (MCGs) and agreements within the MCGs usually contain financial incentives to deliver care [31]. These incentives might differ from the incentives in the contract between the hospital and health insurer. This indicates a possible risk regarding uniformity in policy between the hospital and MCG(s) and might reduce the desired effect of a hospital reimbursement method with little incentives [26]. Therefore, the relation between the reimbursement model and the external distribution model does not ensure required change for appropriate care in financial output.

The external distribution model holds specific Dutch characteristics. One such characteristic is that a (small) majority of physicians are self-employed. In that aspect, the system with self-employed medical specialists in the United States is most similar. Nevertheless, the results regarding contracts with MCGs are applicable to other countries. Even when medical specialists are salaried, there (typically) are incentives to provide care. This relationship is not contracted separately, but is part of the hospital reimbursement model. In this case, the amount of care provided might have an effect on the number of medical specialists employed. Therefore, for both self-employed and salaried medical specialists, providing care is important. The incentive for medical specialists applies equally well in countries where all medical specialists are salaried by the hospital.

### Strengths and limitations

This study has provided a framework to understand the complex financial model of Dutch hospital care. Other healthcare systems are also confronted with gaps between the reimbursement and distribution models. This case study may be relevant for their thoughts on this issue since the Dutch model, thanks to its versatility in contract types and budgeting methods, covers a whole line of possible relations between reimbursement and distribution models. The effect of the distribution model in hospitals is of great importance internationally. Because of the complexity of the funding system and the related cross-subsidization, understanding the financial incentives in the system is of great value. Furthermore, an in-depth economic analysis would be of great value for follow-up research. The high response rate for the questionnaire contributes to the examination of the framework. It might be a limitation restricting the questionnaire to financial experts in Dutch hospitals. However, considering the specific questions, the expert approach seems to generate the most accurate impression of the Dutch situation. Furthermore, the study explores a missing link in the financial model of hospital care, which requires certain practical and theoretical knowledge. It can be argued that perspectives of other stakeholders could have been included, such as health insurers and MCGs. In follow-up research it can indeed be relevant to explore their views and influence. The Covid pandemic might have affected the results since regular contract negotiations were frozen in the past two years. Therefore we requested to choose 2020 as the reference year when answering the questions.

## Conclusions

In an effort to contribute to the research on affordability of hospital care, we examined the internal distribution of resources as a missing link in the hospital financing system. Based on this research in Dutch setting, we indicate a limited interaction between the reimbursement model and the distribution model. To achieve affordable and accessible hospital care, contractual agreements with health insurers contain financial incentives. The lack of congruence between the reimbursement model and the distribution model might limit the desired effects of incentives in contractual agreements aimed at the financial output.

It was not our purpose to identify the most desirable combination between reimbursement and distribution models. However, long-term stable contracts with few financial incentives combined with a flexible budget methodology might be assumed to have the most influence on financial output of hospitals.

To our knowledge, this is the first study focused on the interaction between contractual payer agreements and budget distribution in hospitals. Our results indicate that this interaction might contain valuable insights for reimbursement policies that seek to contribute to the affordability of hospital care. In addition to financial output, the effects on quality of care should ideally be addressed. The extent to which the distribution model shields professionals from the incentives built into the reimbursement model should be further investigated with attention to the behavioral component. Despite international differences in the financing of hospital care, further research on this topic is warranted. A better understanding of the various interactions and incentives as visualized in our conceptual framework, could result in evidence-based advice for achieving affordable and accessible hospital care.

### Supplementary Information

**Additional file 1.** 

**Additional file 2.** 

**Additional file 3.** 

## Data Availability

The dataset generated and analyzed during the current study is not publicly available because it contains confidential and competitively susceptible information. The dataset is available from the corresponding author upon reasonable request.
