# Supplementary material for: Reimbursement of care does not equal the distribution of hospital resources: an explorative case study on a missing link among Dutch hospitals
Source: BMC Health Serv Res. 2023 Sep 19;23:1007. doi: 10.1186/s12913-023-09649-4 (PMC10507878; doi:10.1186/s12913-023-09649-4)
Supplement: Supplementary file 1 — Additional file 1. [file 12913_2023_9649_MOESM1_ESM.docx]

## Supplementary 1: Questionnaire

**Financing structures in Dutch hospitals**

Many thanks for agreeing to participate in this survey. The survey consists of up to 22 questions about your hospital's financing structure, divided into 14 multiple-choice questions and 8 open-ended questions. The questionnaire will take a maximum of 20 minutes to complete and is open until April 1, 2022.

Because of the Covid pandemic, in almost all hospitals both the contracts with health insurers as well as the budget structure have been affected. Therefore, we request to choose **2020** as the reference year when answering the questions. If your contract agreements or budget for 2020 had not yet been finalized when the effects of the Covid pandemic became apparent you can use the year 2019 when answering the questions.

We ensure the data will be treated confidentially. Also, the results will not be traceable at hospital level. If you have any questions regarding this questionnaire or the study to be conducted, please contact me at the e-mail address below. Thank you very much for your cooperation.

Kind regards, also on behalf of

Prof. Dr. Bart Berden

Prof. Dr. Patrick Jeurissen

Dr. Roos Mesman

Lisanne van Leeuwen

PhD candidate Radboud University

Lisanne.VL.vanLeeuwen@radboudumc.nl

**General questions**

| **Question** | **Response options** | **Mandatory** | **Adjusted next question** |
| --- | --- | --- | --- |
| 1. Name | Open field | No |  |
| 2. Job title | Open field | No |  |
| 3. Email | Open field | No |  |
| 4. Name of the hospital where you work | Open field | No | If empty, go to question 5. Otherwise, go to question 7. |

**Hospital characteristics**

| **Question** | **Response options** | **Mandatory** | **Adjusted next question** |
| --- | --- | --- | --- |
| 5. The type of hospital for which you are completing the  questionnaire | 1. General hospital 2. Teaching hospital 3. University Medical Center | Yes | Answer c: question 7, 18a, and 18b are skipped. |
| 6. What is the total contract size with the health insurers? | 1. < 200 mln euro 2. 200 - 400 mln euro 3. 400 - 600 mln euro 4. > 600 mln euro | Yes |  |

**Hospital characteristics – employment of medical specialists**

The employment of medical specialists varies in each hospital. In some hospitals, all medical specialists are employed by the hospital. In most Dutch hospitals more than half of medical specialists are united in one or more Medical Consultant Groups (MCGs). For each hospital, the external distribution model (**orange** arrow in figure 1) can be structured differently. For example, multiple MCGs or more specifically Medical Consultant Groups such as a Dental Specialist Consultant Group (DSCG).


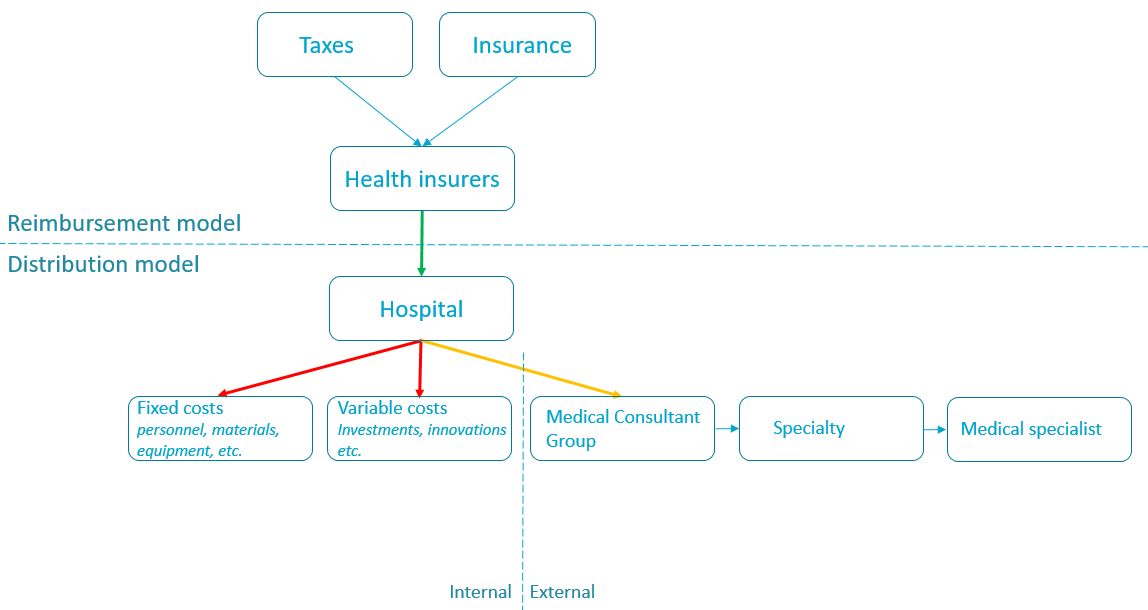


*Figure 1: Conceptual framework of the financial system in hospitals with medical specialists united in a Medical Consultant Group. Medical specialists employed by the hospital are included under "personnel"*

| **Question** | **Response options** | **Mandatory** | **Adjusted next question** |
| --- | --- | --- | --- |
| 7. Are one or more Medical Consultant Groups associated with the hospital? | 1. Yes, one MCG 2. Yes, 2-3 MCGs 3. Yes, more than 3 MCGs 4. No MCGs are associated | Yes | Answer a: question 18b is skipped  Answer b or c: question 18a is skipped  Answer d: question 18a and 18b are skipped |

**Questions related to the budget allocation for healthcare departments in your hospital**

The following questions relate to the **red** arrows as shown in figure 1. The questions are specifically focused on the budget system used in your hospital. In practice, often more than one budgeting system is in use. For the next question, an estimation is asked to what extent the budgeting system is used.


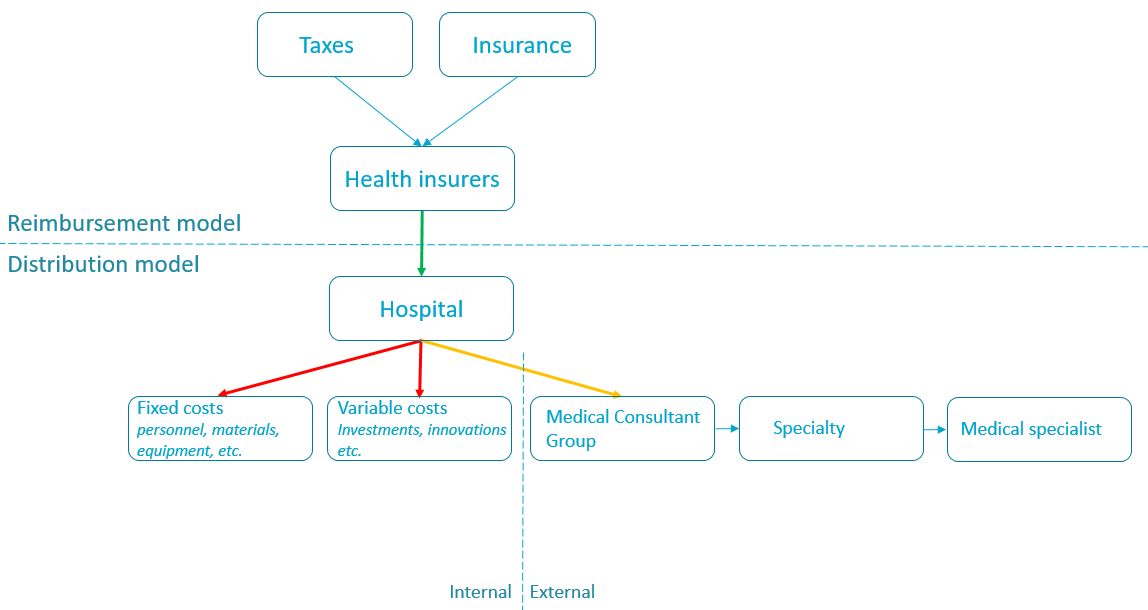


*Figure 1: Conceptual framework of the financial system in hospitals with medical specialists united in a Medical Consultant Group. Medical specialists employed by the hospital are included under "personnel"*

**Activity-based budgeting:** cost budget based on realization in provided care in previous years (cost prices and profiles).

**Incremental budgeting:** budget equal to previous year with correction for indexing and specific changes in provided care.

**Direct budgeting:** direct allocation of budget for (one-time) investments or other specific (one-time) expenses.

| **Question** | **Response options** | **Mandatory** | **Adjusted next question** |
| --- | --- | --- | --- |
| 8a. Rank the budget systems from most used to least used. If a budget system is not used you don’t need to add it. | Rank:   1. Activity-based budgeting 2. Incremental budgeting 3. Direct budgeting 4. Other | No |  |
| 8b. For each budget system, indicate to which extent it is used:  1. Activity-based budgeting  2. Incremental budgeting  3. Direct budgeting  4. Other | a. < 20%  b. 20-39%  c. 40-59%  d. 60-79%  e. > 80%  f. Not applicable | Yes | If budget system “Other” is not applicable (answer f), question 9 is skipped. |
| 9. In case you have chosen 'other budget system', please describe the budget system. | Open field | Yes |  |

**Questions regarding contract agreements with insurers**

The following questions relate to the **green** arrow as shown in figure 1.


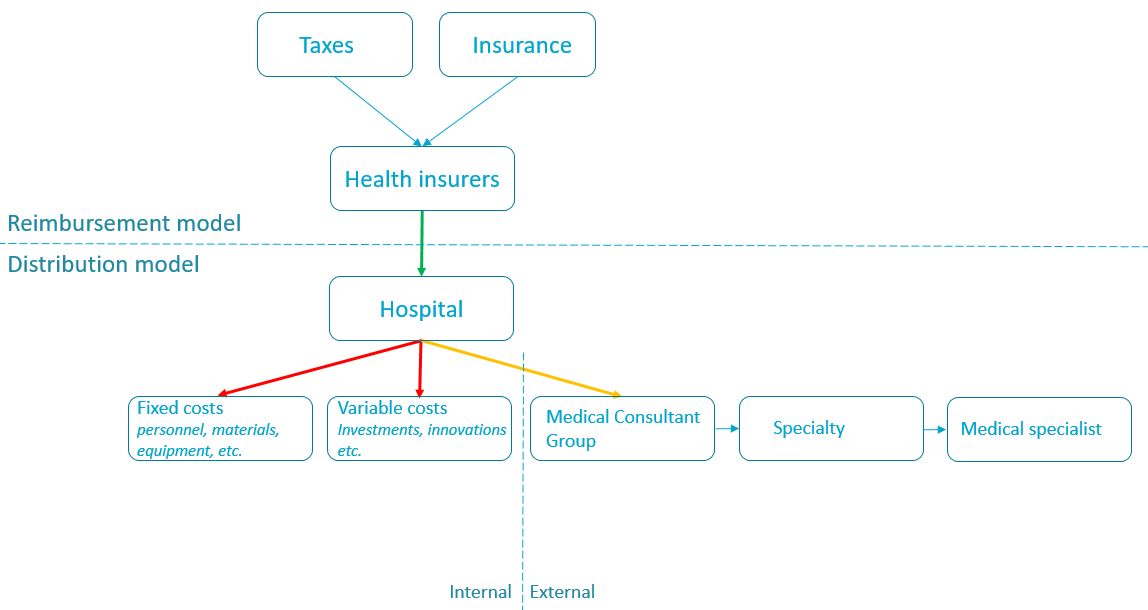


*Figure 1: Conceptual framework of the financial system in hospitals with medical specialists united in a Medical Consultant Group. Medical specialists employed by the hospital are included under "personnel"*

Different types of contracts can be identified with health insurers. The most common contract types are:

**Lump sum agreement:** a fixed contract amount, regardless of realized provided care.

**P*Q agreement with a maximum cap:** provided care is contracted with price and volume. If the cap is reached, additional care is not reimbursed.

**P*Q agreement with adjusted prices above a maximum cap:** provided care is contracted based on price and volume. When reaching the cap, additional care is reimbursed at a reduced rate.

**P*Q agreement without a maximum cap:** provided care is contracted with price and volume without a maximum turnover.

| **Question** | **Response options** | **Mandatory** | **Adjusted next question** |
| --- | --- | --- | --- |
| 10. What is the market share of the largest health insurer? | a. > 60%  b. 51% - 60%  c. 41% - 50%  d. 30% - 40%  e. < 30% | Yes | Answer a or b, go to question 13. |
| 11. What is the combined market share of the largest two health insurers? | a. > 60%  b. 51% - 60%  c. 41% - 50%  d. 30% - 40%  e. < 30% | Yes |  |
| 12. Have you reached similar contract agreements with the two health insurers with the largest market share? | a. Yes  b. No | Yes |  |
| 13. What is the duration of the contract with the health insurer with the largest market share? | a. 1 year  b. 2 year  c. 3 year  d. 4 year  e. 5 year  f. > 5 year | Yes |  |
| 14a. Rank the contract types from most used to least used. If a contract type is not used you don’t need to add it. | Rank:  1. Lump sum agreement  2. P*Q agreement with a maximum cap  3. P*Q agreement with adjusted prices above a maximum cap  4. P*Q agreement without a maximum cap  5. Other | No |  |
| 14b. For each contract type, indicate to which extent it is used:  1. Lump sum agreement  2. P*Q agreement with a maximum cap  3. P*Q agreement with adjusted prices above a maximum cap  4. P*Q agreement without a maximum cap  5. Other | a. < 20%  b. 20-39%  c. 40-59%  d. 60-79%  e. > 80%  f. Not applicable | Yes | If contract type “Other” is not applicable (answer f), question 15 is skipped. |
| 15. In case you have chosen 'other contract type', please describe the contract type. | Open field | Yes |  |

**Questions regarding the relation between contract agreements and the budget system**

| **Question** | **Response options** | **Mandatory** | **Adjusted next question** |
| --- | --- | --- | --- |
| 16. Imagine that in the contract negotiation a 3% price or volume increase is agreed upon. Can you define the way this increase is related to the budget? | a. Direct relations: the entire budget is increased by 3% distributed among all sub-budgets  b. Indirect relation: the budget is increased by more than 3% in some departments and less in others but the entire 3% is incorporated into the budget  c. No relation: the 3% is not (entirely) incorporated into the budget | Yes |  |
| 16a. Can you explain this relation? | Open field | Yes |  |
| 17. Imagine that in the contract negotiation a price or volume increase of 3% is agreed upon for ophthalmology. Can you define the way this increase is related to the budget? | a. Direct relation: ophthalmology's budget is adjusted upward by 3%  b. Indirect relation: ophthalmology's budget is adjusted upward, but not by 3%  c. No relation: ophthalmology's budget is not adjusted based on this specific agreement | Yes |  |
| 17a. Can you explain this relation? | Open field | No |  |
| 18a. Do changes in contract agreements with health insurers affect contract arrangements with the Medical Consultant Group? | a. Yes  b. No | Yes |  |
| 18b. Do changes in contract agreements with health insurers affect contract arrangements with the Medical Consultant Groups? | a. Yes, this affects the contract arrangements with one Medical Consultant Group  b. Yes, this affects contract arrangements with more than one Medical Consultant Group, but not all Medical Consultant Groups  c. Yes, this affects the contract arrangements with all Medical Consultant Groups  d. No, this does not affect the contract arrangements with the Specialist Companies | Yes | Answer c or d: question 19 is skipped. |
| 19. What is the reason for the differentiation between the Medical Consultant Groups? | Open field | Yes |  |

**In conclusion**

| **Question** | **Response options** | **Mandatory** | **Adjusted next question** |
| --- | --- | --- | --- |
| 20. Would you like to be informed about the results of this study? | a. Yes  b. No | Yes | If the answer is a and question 3 is empty: question 20a is shown. Otherwise go to question 21. |
| 20a. Email | Open field | Yes |  |
| 21. May we contact you for possible follow-up research, for example, an interview or focus group discussion? | a. Yes  b. No | Yes | If the answer is a and questions 3 and 20a are empty: question 21a is shown. Otherwise go to question 22. |
| 21a. Email | Open field | Yes |  |
| 22. If you have any questions and/or comments regarding this questionnaire please enter them in the text field below. You can also contact us at Lisanne.vl.vanleeuwen@radboudumc.nl | Open field | No |  |
