# Supplementary material for: Reimbursement of care does not equal the distribution of hospital resources: an explorative case study on a missing link among Dutch hospitals
Source: BMC Health Serv Res. 2023 Sep 19;23:1007. doi: 10.1186/s12913-023-09649-4 (PMC10507878; doi:10.1186/s12913-023-09649-4)
Supplement: Supplementary file 2 — Additional file 2. [file 12913_2023_9649_MOESM2_ESM.docx]

## Supplementary 2: Tables

*Table 1: Overview of the study sample*

|  | **Dutch hospitals in 2022** | | **Approached hospitals** | | **Participating hospitals** | | **Participating / Total** | **Participating / Approached** |
| --- | --- | --- | --- | --- | --- | --- | --- | --- |
| **University Medical Center** | **7** | **11%** | **5** | **10%** | **5** | **13%** | **71%** | **100%** |
| Advisor (finance) |  |  |  |  | 1 |  |  |  |
| Controller |  |  |  |  | 1 |  |  |  |
| Manager (finance, control and/or sales) |  |  |  |  | 3 |  |  |  |
| **Teaching hospital** | **27** | **43%** | **27** | **55%** | **24** | **63%** | **89%** | **89%** |
| Advisor (finance) |  |  |  |  | 2 |  |  |  |
| Controller |  |  |  |  | 2 |  |  |  |
| Director |  |  |  |  | 3 |  |  |  |
| Manager (finance, control and/or sales) |  |  |  |  | 17 |  |  |  |
| **General hospital** | **29** | **46%** | **17** | **35%** | **9** | **24%** | **31%** | **53%** |
| Manager (finance, control and/or sales) |  |  |  |  | 9 |  |  |  |
| **Total** | **63** | **0** | **49** | **0** | **38** | **0** | **60,3%** | **77,6%** |

*Note: Due to differences in the organizational structure of hospitals, job titles do not always correspond*

*Table 2: Number of MCGs*

|  | **University Medical Center** | **Teaching hospital** | **General hospital** | **Total** |
| --- | --- | --- | --- | --- |
| No Medical Consultant Groups | 5 | 1 | 1 | 7 |
| 1 Medical Consultant Group |  | 15 | 1 | 16 |
| 2-3 Medical Consultant Groups |  | 6 | 7 | 13 |
| > 3 Medical Consultant Groups |  | 2 |  | 2 |
| **Total** | **5** | **24** | **9** | **38** |
