# Supplementary material for: Reimbursement of care does not equal the distribution of hospital resources: an explorative case study on a missing link among Dutch hospitals
Source: BMC Health Serv Res. 2023 Sep 19;23:1007. doi: 10.1186/s12913-023-09649-4 (PMC10507878; doi:10.1186/s12913-023-09649-4)
Supplement: Supplementary file 3 — Additional file 3. [file 12913_2023_9649_MOESM3_ESM.docx]

## Supplementary 3: Figures

*Figure 2: Use of contract types, ordered by level of incentives (high to low)*

*Figure 3: Use of budget methods, ordered by level of flexibility (high to low)*
